# The Epithelial Egg Tooth of the Chicken Shares Protein Markers with the Embryonic Subperiderm and Feathers

**DOI:** 10.3390/jdb14010001

**Published:** 2025-12-22

**Authors:** Attila Placido Sachslehner, Julia Steinbinder, Claudia Hess, Veronika Mlitz, Leopold Eckhart

**Affiliations:** 1 Department of Dermatology, Medical University of Vienna, 1090 Vienna, Austria; attila.sachslehner@meduniwien.ac.at (A.P.S.);; 2 Clinic for Poultry and Fish Medicine, Department for Farm Animals and Veterinary Public Health, University of Veterinary Medicine Vienna, 1210 Vienna, Austria

**Keywords:** skin appendages, egg tooth, feathers, keratinocytes, differentiation, cornification, keratin, periderm, hatching, birds

## Abstract

The epithelial egg tooth is used by birds to open the eggshell for hatching. This ectodermal structure consists of a multilayered periderm and a hard cornified portion, the caruncle or actual egg tooth. Here, we determined the protein composition of the egg tooth of the chicken and compared the proteins to markers of other epithelia identified in previous studies. The egg tooth and the upper beak of chicken embryos of Hamburger and Hamilton (HH) stage 44 were subjected to mass spectrometry-based proteomics. We found that scaffoldin, a marker of the embryonic periderm and the feather sheath, was enriched in the egg tooth relative to the beak. Likewise, Epidermal Differentiation protein containing DPCC Motifs (EDDM) and Epidermal Differentiation protein starting with a MTF motif and rich in Histidine (EDMTFH), which had previously been characterized as markers of the subperiderm on embryonic scutate scales and the barbs of feathers, were also enriched in the egg tooth. The expression of EDDM and EDMTFH was confirmed RT-PCR analysis. Our data suggest that the epithelial egg tooth is related to the subperiderm and feathers, a hypothesis with potentially important implications for the evolution of the avian integument.

## 1. Introduction

Epithelial cells of the skin form the epidermis and skin appendages such as hair, nails and feathers [1,2,3,4,5]. During embryonic development a specialized epithelial layer, known as the periderm, is present on the surface and prevents fusion of physically adjacent epithelia [6,7,8,9,10,11,12]. The epithelia of the skin comprise stem cells, proliferating cells, also referred to as transit-amplifying cells, and differentiating cells. Cell differentiation involves the expression of genes for proteins that facilitate specialized functions, such as the secretion from glandular epithelia, establishment of a barrier against the environment in the epidermis, and the establishment of mechanically resilient and durable skin appendages such as hair shafts, nails, feathers, beaks and scales [13,14,15]. Differentiation towards hard skin appendages, epidermal keratinocyte differentiation and late differentiation of the embryonic periderm result in cornification, which is a mode of programmed cell death involving extensive cross-linking of proteins [15]. Cornified epithelial structures can be isolated and their protein composition can be determined by mass spectrometry-based proteomic analysis [16,17].

The epithelial egg tooth is an evolutionarily ancient skin appendage of amniotes which is used to mechanically open the egg and thereby facilitates hatching [18]. It forms on the upper jaw during embryonic development of birds, crocodilians, turtles and monotremes [19,20,21,22,23,24]. Squamate reptiles develop a mineralized egg tooth homologous to teeth of adult amniotes [25]. The epithelial egg tooth is likely to have evolved in a common ancestor of all amniotes, which depended on embryonic development in an egg with a mechanically resilient shell, and to have been lost in squamates, where a mineralized egg tooth substitutes for it, and in therian mammals, which develop without incubation in an egg [24]. Thus, in addition to the appearance of an efficient skin barrier against a dry environment, the epithelial egg tooth was a critical evolutionary innovation of stem amniotes that was modified or lost when subgroups of amniotes further adapted to life on land.

The egg teeth of birds, crocodiles and turtles and its equivalent, known as the caruncle, of the platypus, a protherian mammal, consist of epithelial cells that differentiate and undergo cornification. The molecular control of egg tooth growth and differentiation is only incompletely known [26,27,28]. Retinoic acid and Noggin-mediated inhibition of bone morphogenetic protein signaling are able to trigger ectopic egg tooth development [26]. The most detailed studies of the histology and gene expression of the egg tooth have been performed in the chicken [29]. The chicken egg tooth starts to develop at HH stage 30 around day 6–7 of embryonic development [30]. It reaches its final size around HH 40 (embryonic day 14). Within 1 or 2 days after hatching, the egg tooth falls off [30]. Histologically, the egg tooth is located on top of the epithelium destined to form the epithelial compartment of the upper beak, also known as the rhamphotheca. Like the skin on other body parts, this site is covered by a periderm which can be considered as the outer compartment of the developing egg tooth [31]. The periderm of the egg tooth is markedly thickened in comparison to the other periderm, as it consists of multiple cell layers [29,32]. Characteristically, it contains so-called periderm granules which are visible in histological sections and electron microscopy [32]. Beneath the periderm, the actual egg tooth develops. In contrast to the periderm, which disintegrates in the final stages of development, the proper egg tooth hardens and remains firmly attached to the tissue underneath so that it can be used to rupture the eggshell in the course of hatching.

The cell differentiation program of the egg tooth and the molecular composition of the proper egg tooth have remained largely unknown. The peridermal compartment of the egg tooth contains the same marker protein, scaffoldin, as the regular periderm [29,33]. Scaffoldin is also expressed in the sheath of feather follicles, suggesting a role similar to its mammalian homolog, trichohyalin, which is expressed in the inner root sheath of hair follicles [32,33,34]. Scaffoldin and trichohyalin are encoded by genes located within a gene cluster known as the epidermal differentiation complex (EDC) [35,36,37,38,39,40,41]. Corneous beta-proteins (CBPs) are expressed in the egg tooth of birds and turtles [23,32]. Recently, transglutaminase 9 (TGM9) was reported to be expressed in the chicken egg tooth [42], suggesting that TGM-dependent isopeptide-bonds between proteins contribute to cornification of the egg tooth.

The aim of the present study was to determine the protein composition of the chicken egg tooth and to compare it to the proteomes of other epithelia. The data were used to build a new hypothesis about the evolutionary trajectory of skin appendages in birds.

## 2. Materials and Methods

### 2.1. Preparation of Egg Tooth and Beak Samples

Egg tooth and beak samples were prepared from stage HH44 chicken embryos as described previously [42]. In brief, the beaks of the chicken embryos were dissected and dried for 3–5 min at room temperature. The egg tooth turned white through the drying step and could be removed from the beak with a pointed scalpel. The epithelial compartment of the upper beak was separated from the underlying tissue with forceps. The samples were frozen in liquid nitrogen and stored at −80 °C. Subsequently, the samples were placed in 200 µL lysis buffer containing 30 mM Tris HCl, 7 M urea (catalog number: 0568, VWR, Radnor, PA, USA), 2 M thiourea (catalog number: T7875, Sigma-Aldrich, St. Louis, MO, USA) and 4% CHAPSO (catalog number: 28304, Pierce, Waltham, MA, USA). Dithiothreitol was added to the samples at a final concentration of 0.2 M, and the samples were incubated at 70 °C for 3 h. Subsequently, the samples were homogenized with a Precellys homogenizer (VWR) using lysis tubes filled with beads (catalog number: 31-00589, IST Innuscreen GmbH, Berlin, Germany). After centrifugation at 18,000× *g* for 15 min at 4 °C, the supernatant was collected, and the pellet was subjected to sonication using an Hielscher Ultrasound Technology sonicator (amplitude 100, two times 30 s). The solution was centrifuged and the resulting supernatant was pooled with the supernatant of previous homogenization step. Supernatants were stored at −80 °C [39].

For each sample, the protein lysate was topped up to 500 µL with 8 M urea (catalog number: 2317.1, Roth, Karlsruhe, Germany) in 50 mM Tris and loaded onto a Pall 10 kD filter according to the filter aided sample preparation (FASP) method [43]. The solution was centrifuged twice for 20 min at 10,000× *g*. The cysteine residues of the proteins were reduced with 200 mM dithiothreitol (37 °C, 30 min, catalog number: 6908.3, Roth) and alkylated with 500 mM iodoacetamide (catalog number: I1149, Sigma-Aldrich) at 37 °C for 30 min on the filter [42].

### 2.2. Liquid Chromatography–Mass Spectrometry

Before liquid chromatography–mass spectrometry (LC-MS) analysis, peptide extracts were desalted and cleaned up using C18 spin columns (catalog number: 89870, Thermo-Fisher, Waltham, MA, USA) according to the manufacturer’s protocol. The dried peptides were dissolved in 15 µL 0.1% trifluoroacetic acid (catalog number: 10723857, Thermo-Fisher). Peptides were separated on a nano-HPLC Ultimate 3000 RSLC system (Dionex). Sample pre-concentration and desalting was accomplished with a 5 mm Acclaim PepMap μ-Precolumn (300 µm inner diameter, 5 µm particle size, and 100 Å pore size) (Dionex, Sunnyvale, CA, USA). For sample loading and desalting, 2% acetonitrile in ultra-pure LC-MS grade water with 0.05% trifluoroacetic acid was used as a mobile phase with a flow rate of 5 µL/min. Separation of peptides was performed on a 25 cm Acclaim PepMap C18 column (75 µm inner diameter, 2 µm particle size, and 100 Å pore size, OD010C34, Pall, Port Washington, NY, USA) with a flow rate of 300 nL/min. The gradient started with 4% mobile phase B (80% acetonitrile with 0.08% formic acid, catalog number: 28905, Pierce) for 7 min, increased to 31% in 60 min and to 44% in additional 5 min. This was followed by a washing step with 95% phase B. Mobile phase A consisted of ultrapure LC-MS grade H_2_O with 0.1% formic acid. For mass spectrometric analysis, the LC was directly coupled to a high-resolution Q Exactive HF Orbitrap mass spectrometer.

MS full scans were performed in the ultrahigh-field Orbitrap mass analyzer in ranges m/z 350−2000 with a resolution of 60,000, the maximum injection time (MIT) was 50 ms and the automatic gain control (AGC) was set to 3 × 10^6^ The top 10 intense ions were subjected to Orbitrap for further fragmentation via high energy collision dissociation (HCD) activation over a mass range between m/z 200 and 2000 at a resolution of 15,000 with the intensity threshold at 4 × 10^3^ Ions with charge states +1, +7, +8 and >+8 were excluded. Normalized collision energy (NCE) was set at 28. For each scan, the AGC was set at 5 × 10^4^ and the MIT was 50 ms. Dynamic exclusion of precursor ion masses over a time window of 30 s was used to suppress repeated peak fragmentation.

### 2.3. Protein Identification

The MS data were subjected to an initial analysis that was published previously [39]. For the present study, the data were re-analyzed according to the following approach. The reference protein sequence database included proteins predicted in the chicken genome assembly GalGal5 and proteins predicted in previous studies of the EDC [44], keratins [45] and transglutaminases [42]. The database search was performed using the Proteome Discoverer Software 2.4.01.30515 (Thermo Fisher Scientific), which calculates protein abundances for each protein based on the abundances of the confidently identified peptide groups. The following parameters were set: RefSeq entries: 129993 (*Gallus gallus*, NCBI Taxonomy ID: 9031), downloaded on 02.04.2025 (NCBI), - Enzyme name: Trypsin (full), - Max. missed cleavage sites: 2, - Precursor mass tolerance: 10 ppm, - Fragment mass tolerance: 0.02 Da, - Dynamic modification: Oxidation/+15.995 Da (M), - Dynamic modification: Deamidation/+0.984 Da (N,Q), - N-terminal modification: Gln->pyro-Glu/−17.027 Da (Q), - N-terminal modification: Acetyl/+42.011 Da (N-Terminus), - N-terminal modification: Met-loss/−131.040 Da (M), - N-terminal modification: Met-loss+Acetyl/−89.030 Da (M), - Static modification: Carbamidomethyl/+57.021 Da (C), Decoy Database search: - Target false discovery rate (FDR) (strict): 0.01, - Target FDR (relaxed): 0.05, - Validation based on: q-Value. Evaluation of protein abundances including normalization to total peptide amount was performed in Proteome Discoverer software (version 2.4.01.30515, Thermo Fisher Scientific, Waltham, MA, USA).

### 2.4. Statistical Analysis of Protein Abundances

The limma package (version: 3.58.1) [46] for R (version 4.3.3), was used for statistical analysis. Limma calculates linear regressions for each protein, and shares information among samples on a global scale to improve data robustness, and variance estimations, respectively. An empirical Bayes moderated t-statistics was performed to assess statistical significance, followed by a multiple testing correction, implemented in limma, according to Benjamini–Hochberg [47]. A beak sample originally termed “beak 1” was excluded from the statistical analysis because the protein profile indicated the aberrant presence of sub-dermal tissue in this sample. Only proteins with at least three detected abundances per sample were included in the limma analysis.

### 2.5. RT-PCR

Isolation of RNA and reverse transcription into cDNA has been reported previously for the Hamburger and Hamilton stages 34–36 [42] which corresponds to 8–10 days of incubation in the egg. Additional cDNAs, sampled in a previous study [29], were re-used and correspond to the Hamburger and Hamilton stages 36, 40, and 44. The following primer pairs (Microsynth, Balgach, Switzerland) were used: chicken *EDDM* (forward: 5′-CGTTAGCTTGTTCCTGGTGG-3′, reverse: 5′-CCTGAACACATGTGCAGACC-3′), chicken *EDMTFH* (forward: 5′-AGCTTCATTCGCTTCTCTCG-3′, reverse: 5′-GCCTGAATGACCACCGATTC-3′), chicken *KRT9LC3* (forward: 5′-CTTGGACAAAGTACGGCTGC-3′, reverse: 5′-TGAAGACTTCCGAGCCAAGT-3′) and chicken *EEF1A1* (forward: 5′-GCCCCGAAGTTCCTGAAATC-3′, reverse: 5′-GGCCTTGATGACACCAACAG-3′). The PCR was run on a T100 thermocycler (Bio-Rad, Hercules, CA, USA) and involved an initial denaturation step at 95 °C for 2 min, 35 cycles at 95 °C for 30 s, 60 °C for 30 s and 72 °C for 20 s, and final extension at 72 °C for 10 min.

## 3. Results

### 3.1. The Egg Tooth and the Beak Epithelium Differ with Respect to Their Protein Composition

**Figure 1:**
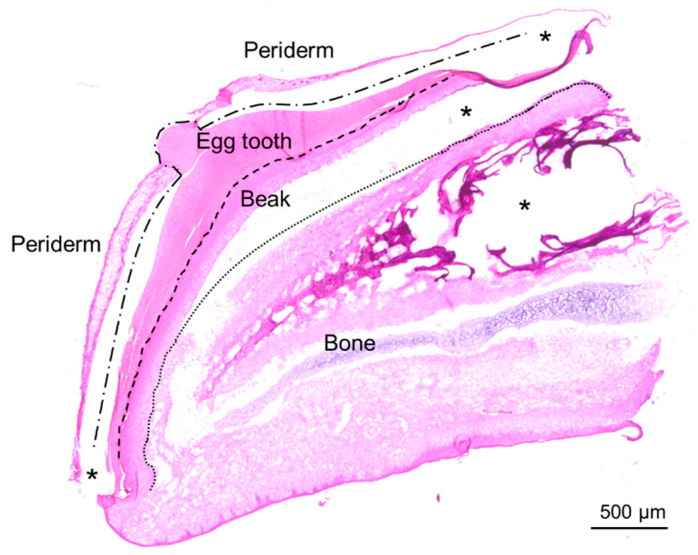
The egg tooth is an epithelial protrusion on the embryonic beak. A section through an upper beak of a chicken at embryonic stage HH44 was stained with hematoxylin and eosin. The compartments of the epithelium are separated by lines. Asterisks indicate open spaces that have appeared during the histological processing of the tissue sample.

The egg tooth of chicken embryos at Hamburger and Hamilton (HH) stage 44 [30] was investigated by histology and proteomic analysis. Hematoxylin and eosin (H&E) staining showed that the egg tooth consisted of two compartments, namely the external periderm and the internal hard egg tooth, also referred to as proper egg tooth (Figure 1).

**Figure 2:**
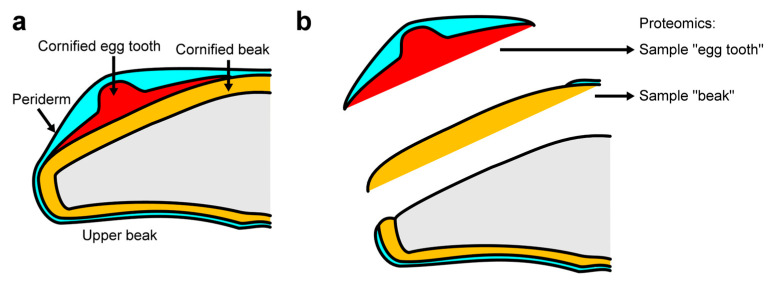
Schematic depiction of tissue sampling for proteomic analysis. (**a**) Schematic depiction of the upper beak and the egg tooth. (**b**) Separation of the egg tooth from the beak and preparation of the beak sample. Brief drying of the tissue made the egg tooth macroscopically visible as a white protrusion, so that it could be detached using a scalpel. The beak sample was cut off distally to the nostrils.

To determine the proteome of the epithelial egg tooth of chicken embryos, the embryos were isolated and the egg tooth was made macroscopically visible by drying the tissue at ambient air for 3–5 min. The egg tooth was mechanically separated from the beak epithelium, and the upper beak was separated from the tissue underneath (Figure 2). Both the egg tooth and the beak samples were investigated further so that proteins enriched in the egg tooth could be identified. Egg tooth and beak samples from four chicken embryos were subjected to proteomic analysis (PRoteomics IDEntifications Database, PRIDE, accession number: PXD048875 [42]). The data from one beak sample were excluded from further comparisons because of aberrant sampling of subdermal tissue, so that the protein abundance data from three beak and four egg tooth samples could be included in the subsequent statistical analysis.

**Figure 3:**
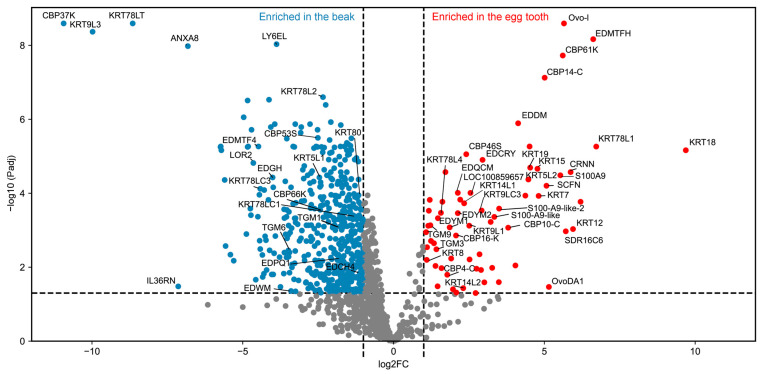
Comparison of protein abundances in the beak and the egg tooth of chicken embryos. Protein abundances in the egg tooth (*n* = 4) and the beak (*n* = 3) of chicken embryos at development stage HH44 were compared. Statistical significance (−log10 of the adjusted *p*-value, Padj) is plotted against the log2 fold change (log2FC). Blue and red dots indicate a −log10 (Padj) of >1.3, corresponding to Padj < 0.05, and a log2FC of <−1 and >1, respectively. Full names of EDC proteins are provided in Table 1.

For quantitative comparison, we used abundance values of proteins (*n* = 1233) that met the criteria for the limma analysis as described in the Section 2. Significant differences in abundances (log2FC < −1 or >1, −log10 Padj > 1.3 equivalent to Padj < 0.05) were detected for 537 and 76 proteins enriched in the beak and egg tooth, respectively (Figure 3, Table S1). The complete list of peptides and proteins identified in the samples is provided in Table S2.

The proteomic analysis identified structural proteins (for details, see the next section) and non-structural proteins presumably regulating cell differentiation and the metabolism of the egg tooth. Among the non-structural proteins, protein cross-linking enzymes such as TGM3 and TGM9 were enriched in the egg tooth, whereas TGM1 and TGM6 were enriched in the beak. The egg tooth also contained high levels of protein-arginine deiminase type-1 (PADI1), which converts arginine into citrulline residues [48], and epidermal retinol dehydrogenase 2, also known as short chain dehydrogenase/reductase family 16C member 6 (SDR16C6) [49]. V-set and immunoglobulin domain-containing protein 8, a functionally uncharacterized protein homologous to a mammalian hair and nail protein [50] (Table S1).

### 3.2. EDC Proteins Are Differentially Expressed in the Egg Tooth and the Beak

The egg tooth contained members of the three main groups of structural proteins of avian epithelia, namely, keratins (KRTs), corneous beta proteins CBPs, which are also known as beta-keratins, and EDC proteins, here defined as proteins encoded by genes of the EDC excluding CBPs (Table S1). Among keratins, KRT18 and KRT78L1 were most strongly enriched in the egg tooth. Among cysteine-rich keratins, which are implicated in hard cornification due to their ability to form intermolecular disulfide bonds [45,51], KRT9LC3 was enriched in the egg tooth (Table S1). CBP61-K and CBP14-C were enriched in the egg tooth, whereas CBP37K was enriched in the beak (Table S1).

Our investigation was focused on EDC proteins because some of these proteins had been characterized as markers of different epithelial structures of the chicken [33,37,52,53]. Both S100 fused-type (SFTP) proteins of the EDC, namely cornulin (CRNN) and scaffoldin (SCFN), an ortholog of mammalian trichohyalin [33], were enriched in egg tooth samples (Table 1). This finding is in line with the detection of these proteins in the multi-layered periderm portion of the egg tooth [29,32,33]. Among proteins encoded by single-coding exon EDC (SEDC) genes [37], EDDM and EDMTFH were the most strongly enriched. These two proteins were previously shown by immunohistochemistry as markers of both the subperiderm of embryonic scutate scales and barbs or barbules of feathers [52,53]. Furthermore, EDCRY, EDQCM, EDYM2 and EDYM1 were enriched in the egg tooth, whereas EDPQ1, EDWM, EDGH, EDMTF4 and LOR2 were enriched in the beak (Table 1).

**Table 1:** Expression of EDC proteins in the egg tooth in comparison to the beak.

Name (Abbreviation)	Description	Ratio *: Egg Tooth/Beak	Padj	Expression Sites Detected in Previous Studies	References
EDMTFH	epidermal differentiation protein starting with MTF motif and rich in histidine	98	6.80 × 10^−9^	subperiderm, feathers (protein); E18 skin, feathers, scales (mRNA)	[37,53]
CRNN	Cornulin	58	2.68 × 10^−5^	periderm, subunguis (protein)	[33]
SCFN	Scaffoldin	34	6.27 × 10^−5^	periderm, subunguis, feather sheath (protein)	[32,33,54]
EDDM	epidermal differentiation protein containing DPCC motifs	18	1.28 × 10^−6^	subperiderm, feathers (protein); E18 feathers, scales (mRNA)	[37,52,54]
EDCRY	epidermal differentiation protein rich in cysteine, arginine and tyrosine	7.7	1.25 × 10^−5^	n.d.	n.a.
EDYM2	epidermal differentiation protein containing Y motif 2	4.4	3.45 × 10^−4^	E18 scales, beak, skin (mRNA)	[37]
EDQCM	epidermal differentiation protein containing glutamine cysteine motifs	4.4	9.75 × 10^−5^	feathers, claws; E18 beak, scales, skin (mRNA)	[37,54]
EDYM1	epidermal differentiation protein containing Y motif 1	3.6	8.37 × 10^−4^	E18 scales and beak (mRNA)	[37]
EDCH4	epidermal differentiation protein rich in cysteine and histidine 4	0.46	1.44 × 10^−2^	E18 scales, feathers (mRNA)	[37]
EDPQ1	epidermal differentiation protein rich in proline and glutamine 1	0.30	6.08 × 10^−3^	n.d.	n.a.
EDWM	epidermal differentiation protein containing a tryptophan motif	0.095	4.40 × 10^−2^	E18 scales, beak (mRNA)	[37]
EDGH	epidermal differentiation protein rich in glycine and histidine	0.062	3.74 × 10^−5^	n.d.	n.a.
EDMTF4	epidermal differentiation protein starting with an MTF motif 4	0.045	5.43 × 10^−6^	E18 skin, feathers, scales (mRNA)	[37,54]
LOR2	loricrin 2	0.040	1.52 × 10^−5^	skin, skin appendages (mRNA)	[37]

Notes: n.a., not applicable; n.d., not determined; Padj, adjusted *p*-value. * Ratio of protein abundances according to mass spectrometry-based proteomic analysis.

**Figure 4:**
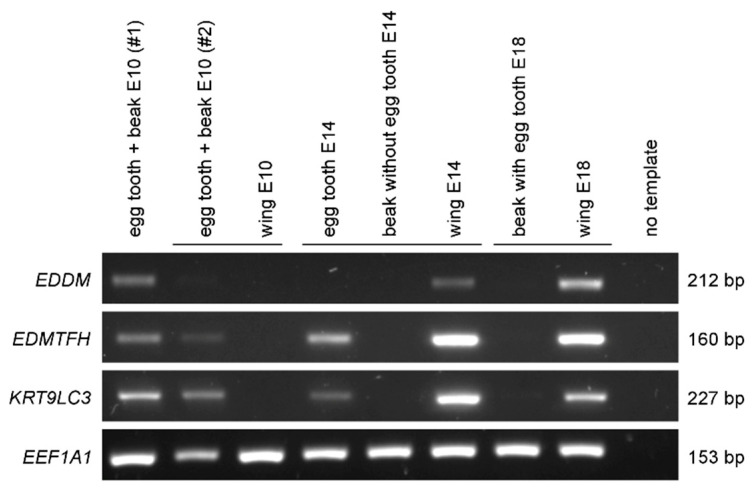
RT-PCR analysis of gene expression during the development of the egg tooth and the beak. *EDDM*, *EDMTFH* and *KRT9LC3* were amplified by RT-PCR in the indicated tissues. PCR products were electrophoresed through an agarose gel. The house-keeping gene *EEF1A1* was used as a control. The size of the PCR products is indicated on the right. Uncropped images of the electrophoresis gel are shown in Figure S3. E, embryonic day; bp, base pairs.

To test whether the proteomic detection of the subperiderm markers EDDM and EDMTFH and of the uncharacterized keratin KRT9LC3 is associated with the presence of the corresponding mRNAs, we performed RT-PCRs with transcript-specific primers. The three genes displayed a similar expression pattern in embryonic tissues. On embryonic day E10, *EDDM*, *EDMTFH* and *KRT9LC3* mRNAs were detected in the egg tooth and beak but not in the wing (Figure 4 and Figure S1). Later in development, these three genes were not expressed in the beak, whereas their mRNAs appeared in the wing, which correlates with the appearance of feather follicles [53]. *EDMTFH* and *KRT9LC3* were also detected in an egg tooth sample which could be separated from the beak of an E14 embryo [33] and *EDDM* mRNA was at the detection limit in this sample, whereas none of the three genes was expressed in the isolated E14 beak (Figure 4). RT-PCR analysis of egg tooth samples from E8 until E10 showed that the expression of *EDDM*, *EDMTFH* and *KRT9LC3* started on day E9 (Figures S1 and S2). Together, the RT-PCRs confirmed expression of *EDDM*, *EDMTFH* and *KRT9LC3* in the egg tooth, which is in agreement with the results of the proteomic analysis (Figure 3, Table S1).

## 4. Discussion

The egg tooth is an important but under-investigated skin appendage of birds. It is critical for hatching of birds and represents an essential evolutionary innovation of amniotes, which has facilitated the transition to fully terrestrial life. Here, we analyzed the chick egg tooth by mass spectrometry-based proteomics using an amino acid sequence reference dataset from GenBank that was extended by sequence predictions of EDC proteins, keratins and transglutaminases [42,44,45]. This allowed us to detect proteins which have been characterized as markers of alternative epithelial cell differentiation pathways [52,53], thereby allowing us to uncover a previously unrecognized compositional similarity of the egg tooth with the subperiderm and likely also with feathers. In addition, the resulting dataset contains many further protein identifications which are available for in depth characterization in follow-up studies.

Among the proteins with the highest enrichment in the egg tooth, there were four EDC proteins, namely CRNN, SCFN, EDMTFH and EDDM. CRNN and SCFN are known to be expressed in the periderm [29,32,33]. Their enrichment is explained by the thickening of the peridermal compartment of the egg tooth. Both CRNN and SCFN contain an S100 domain and a carboxy-terminal domain of low sequence complexity [33]. Like other members of the SFTP family [29], they interact with keratin filaments and contribute to cytoskeletal remodeling in the course of cornification [33]. Post-translational modification of SCFN was suggested by the detection of two Western blot bands [33]. Our data show that PADI1, which catalyzes citrullination of arginine residues, is co-enriched with SCFN in the egg tooth. Given that PADI-dependent modification of the SCFN ortholog trichohyalin occurs in the inner root sheath of hair follicles [48], PADI1 may also modify SCFN in the egg tooth.

The expression and enrichment of EDDM and EDMTFH in the egg tooth is particularly interesting, because both proteins have previously been shown to be expressed specifically in the subperiderm and barbs and barbules of feathers. In addition to belonging to the proteins most enriched in the egg tooth relative to the adjacent beak tissue (Figure 3, Table S1), EDDM and EDMTFH were also confirmed to be expressed by RT-PCR (Figure 4). EDDM contains a high number of cysteine residues implicated in disulfide bond-mediated protein cross-linking [52], and EDMTFH, originally termed histidine-rich protein or fast protein [55,56], contains many histidine residues potentially involved in pi-stacking protein interactions [57]. A third EDC protein component of the subperiderm and feathers, namely epidermal differentiation cysteine-rich protein (EDCRP) [58], was also enriched in the egg tooth (Table S2). The detection of KRT9LC3, a cysteine-rich keratin, both at the mRNA and protein level in the egg tooth and at the mRNA level in wing tissues at the time of feather development, suggesting that its expression parallels that of the aforementioned EDC proteins. The concerted expression of three cysteine-rich proteins (EDDM, EDCRP and KRT9LC3) suggests that these proteins undergo cross-linking via disulfide bonds in a similar manner as the protein components of feathers and hair shafts [15]. Thus, the morphogenesis of the egg tooth appears to depend on multiple modes of protein interactions, including isopeptide bonds formed under catalysis by transglutaminases [42] and disulfide bonds.

**Figure 5:**
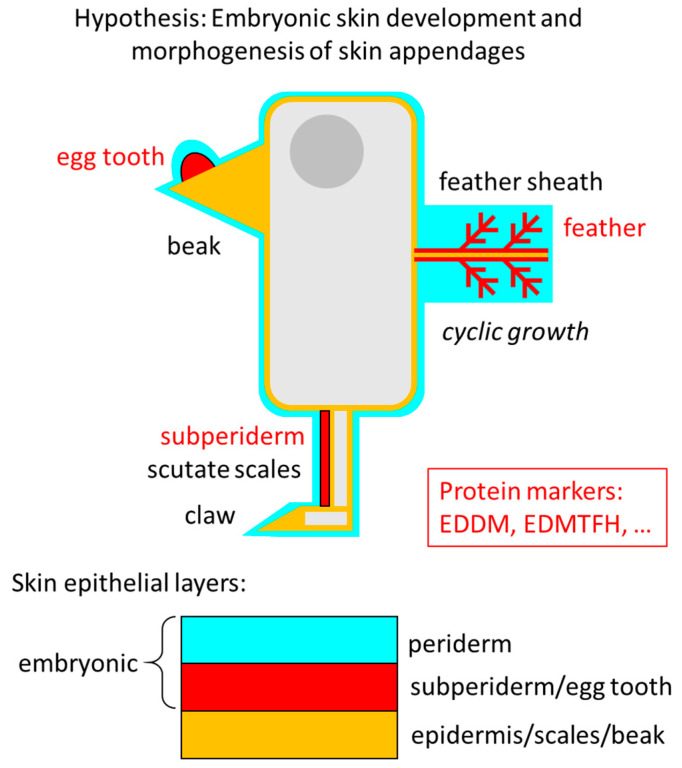
Schematic depiction of the positions of the egg tooth, subperiderm and feathers in a chick embryo. Epithelial layers are highlighted by colors. The periderm covers all epithelia of the skin and skin appendages including the actual egg tooth in the embryo, and it displays similarities to the feather sheath. The subperiderm and the proper egg tooth are located at equivalent positions under the periderm. Common protein markers suggest that the egg tooth is related to the subperiderm on embryonic scutate scales and to the cornifying parts of feathers.

The proper egg tooth is located underneath the periderm and above the epithelial compartment that forms the cornified beak. Its location is homologous to that of the subperiderm, an embryo-specific layer of thick cells between the periderm and the epithelial compartment of hard scutate scales, which are overlapping scales located on the anterior and lateral metatarsus [59,60] (Figure 5). A possible differentiation of subperidermal cells from precursors in the periderm has been discussed [12], but the development and function of the subperiderm have not been fully characterized yet. In a book chapter on the skin of birds, Roger H. Sawyer and co-authors stated that the subperiderm is also “well developed in the epidermis of the embryonic beak” [61]. However, the histological basis for this statement was not included in the book chapter. Thus, we propose that, depending on the terminology, the epithelial egg tooth can be regarded as an equivalent or a subtype of the subperiderm.

Importantly, the detection of feather-associated CBPs/beta-keratins in the subperiderm over scutate scales has led to the hypothesis that feathers are evolutionarily related to the subperiderm [59,60,62]. Subsequently, EDCRP, EDDM and EDMTFH were also detected in both the subperiderm and the feathers, supporting the similarity in protein content and presumably in the protein-dependent molecular architecture of the subperiderm and the feathers [52,53,58]. The results of this study extend this scenario by adding the egg tooth as an expression site of EDCRP, EDDM and EDMTFH. Since the egg tooth has evolved earlier—presumably during the water-to-land transition of stem amniotes—than feathers, we put forward the hypothesis that ancestral egg tooth proteins were coopted to functions in feathers in the phylogenetic lineage leading to birds. Accumulating evidence suggests that avian scutate scales have secondarily evolved from feathers [63,64,65]. Accordingly, shared protein components of feathers, egg tooth and subperiderm are more likely to have originated with functions in the epithelial egg tooth than in the scale-associated subperiderm [59,60,62]. We would like to emphasize that this hypothesis deals only with the evolution of structural proteins of feathers, whereas other aspects of the development, morphogenesis and evolution of feathers have been discussed in other articles [66,67,68,69,70,71,72,73,74,75,76,77,78].

Although we focused on the epithelial egg tooth, this study also provides insights into the composition of the epithelium giving rise to the horny sheath of the beak or rhamphotheca, here referred to as “beak” [79]. For example, we found strong enrichment of two largely uncharacterized proteins, namely CBP37-K, a corneous beta-protein (beta-keratin) of the “keratinocyte” (K) subfamily [44,80], and KRT78LT, a keratin with a particularly long tail domain [45] (Figure 3). The tissue distributions of these proteins have not been determined yet. Considering that the beak is an important research topic in evolutionary-developmental biology [26,79,81,82,83,84], our data may help to identify protein markers for studies of the development and differentiation of the beak in comparison to other cornified structures.

The proteomic analysis of epithelial differentiation products such as the chicken egg tooth and the beak has several advantages relative to other methods. As compared to transcriptomics and other methods based on the detection of mRNAs, proteomics is less dependent on sampling at the right point in time. This is because mRNAs of particular genes are present only transiently during development, whereas many proteins are stably integrated into cornified structures. Furthermore, epithelia undergoing cornification make mRNAs more difficult to extract than proteins which resist harsh lysis conditions. Similarly, mRNA in situ hybridization is hampered by intracellular remodeling during cornification. Accordingly, we could detect several proteins of which the corresponding mRNAs were not found in the developing egg tooth in previous studies [54,85,86].

Nevertheless, the present study has several limitations that necessitate follow-up investigations. First, preparation of the samples “egg tooth” and “beak” was performed by mechanical separation which likely does not exclude minor contaminations by the respective other tissue. Second, the analysis of the proteomic data was designed to quantitatively compare the abundance of individual proteins in the egg tooth and the beak, but not to determine absolute protein abundances. Therefore, the data do not allow the quantitative comparison of different proteins. Third, the proteins were identified in isolated samples of the entire egg tooth without mapping the localization to specific sites within the egg tooth. Immunohistochemical analysis and mRNA in situ hybridization of the egg tooth is difficult because of the hardness of the tissue. In our hands, clear results could only be obtained for TGM9 mRNA [42]. Fourth, we sampled the egg tooth at a single time of development. Data of gene expression from early to late development will require further investigations. Notably, the isolation of the egg tooth is difficult at earlier stages of development whereas in situ localization of gene expression is feasible. Fifth, the hypothesis about the evolutionary-developmental link between the egg tooth, subperiderm and feathers requires more data to be either supported or refuted. More comprehensive comparisons of gene expression in these and other tissues during embryonic development and studies of gene regulation in the spatiotemporal context of development of the chicken are warranted. In addition, the gene expression in the epithelial egg tooth of other species of birds as well as crocodilians, turtles and monotremes are required to explore the evolutionary implications. Further studies should explore these directions of research.

## Data Availability

The dataset analyzed during the current study is available in the PRIDE database, under the accession number PXD048875.

## Supplementary Materials

The following supporting information can be downloaded at: https://www.mdpi.com/article/10.3390/jdb14010001/s1, Figure S1: RT-PCR analysis of gene expression during the development of egg tooth and beak; Figure S2: Uncropped images of electrophoresis gels shown in Figure S1; Figure S3: Whole gel images shown in Figure 4; Table S1: Proteins shown in the volcano plot (Figure 3): Description and statistics; Table S2: Peptides and proteins detected in the egg tooth and beak.
