# Supplementary material for: The Epithelial Egg Tooth of the Chicken Shares Protein Markers with the Embryonic Subperiderm and Feathers
Source: J Dev Biol. 2025 Dec 22;14(1):1. doi: 10.3390/jdb14010001 (PMC12821399; doi:10.3390/jdb14010001)
Supplement: Supplementary file 1 [file jdb-14-00001-s001.zip › Supplementary_Figures.pdf]

Supplementary Figures:

The epithelial egg tooth of the chicken embryo shares protein markers with the subperiderm and feathers

Attila Placido Sachslehner, Julia Steinbinder, Claudia Hess, Veronika Mlitz, Leopold Eckhart

Content:

Figures S1 – S3

Note: Tables S1 and S2 are provided as separate files in Excel format.

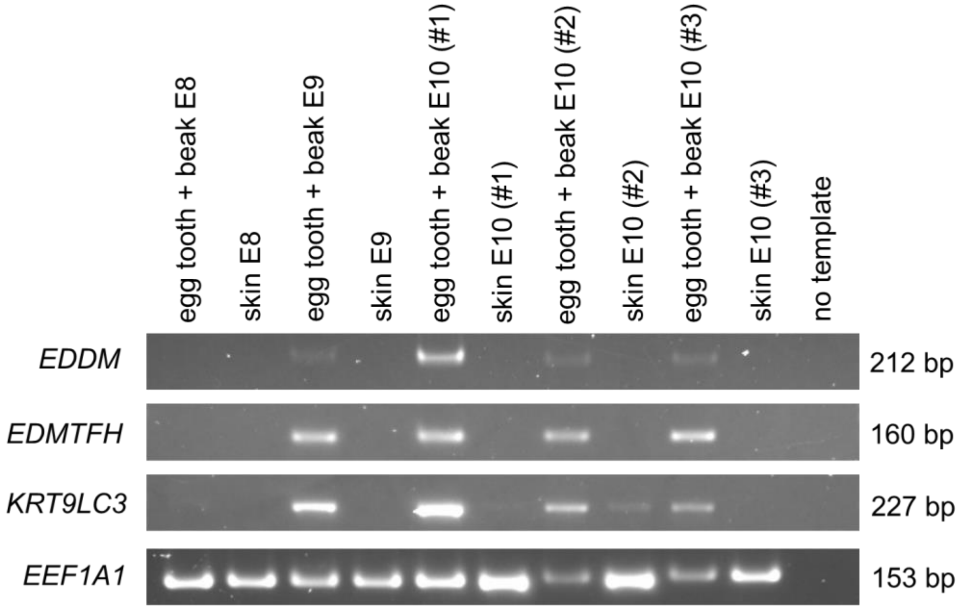

Figure S1. RT-PCR analysis of gene expression during the development of the egg tooth and the beak. Samples comprising the egg tooth and the beak and skin samples were prepared on embryonic days E8, E9 and E10. Three biological replicates (#1, #2, #3) of E10 samples were prepared. The house-keeping gene *EEF1A1* was used as a control. Sizes of PCR products are indicated on the right. bp, base pairs.

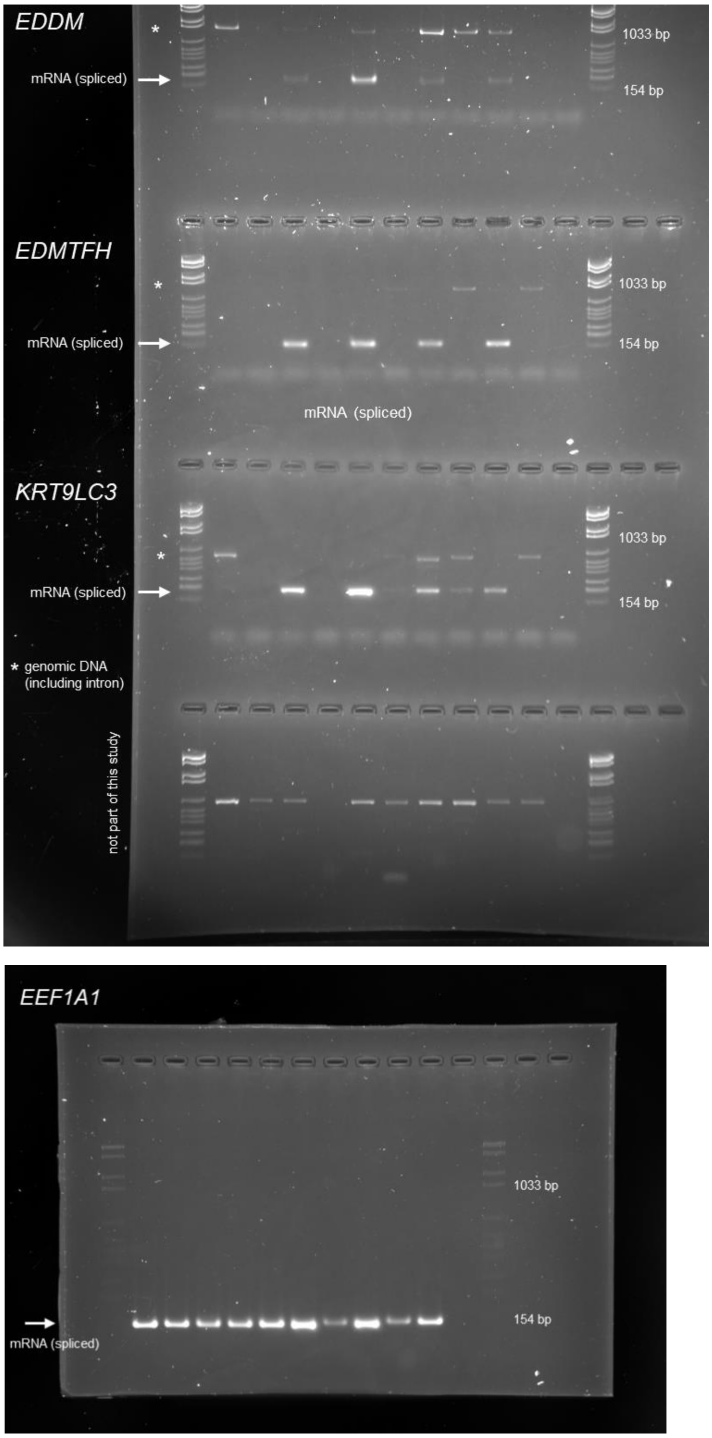

Figure S2. Whole-gel images corresponding to Figure S1. RT-PCR products of *EDDM*, *EDMTFH*, *KRT9LC3* (upper image) and of the house-keeping gene *EEF1A1* (lower image) were separated by agarose gel elecrophoresis. The sizes of two marker bands are indicated on the right. bp, base pairs.

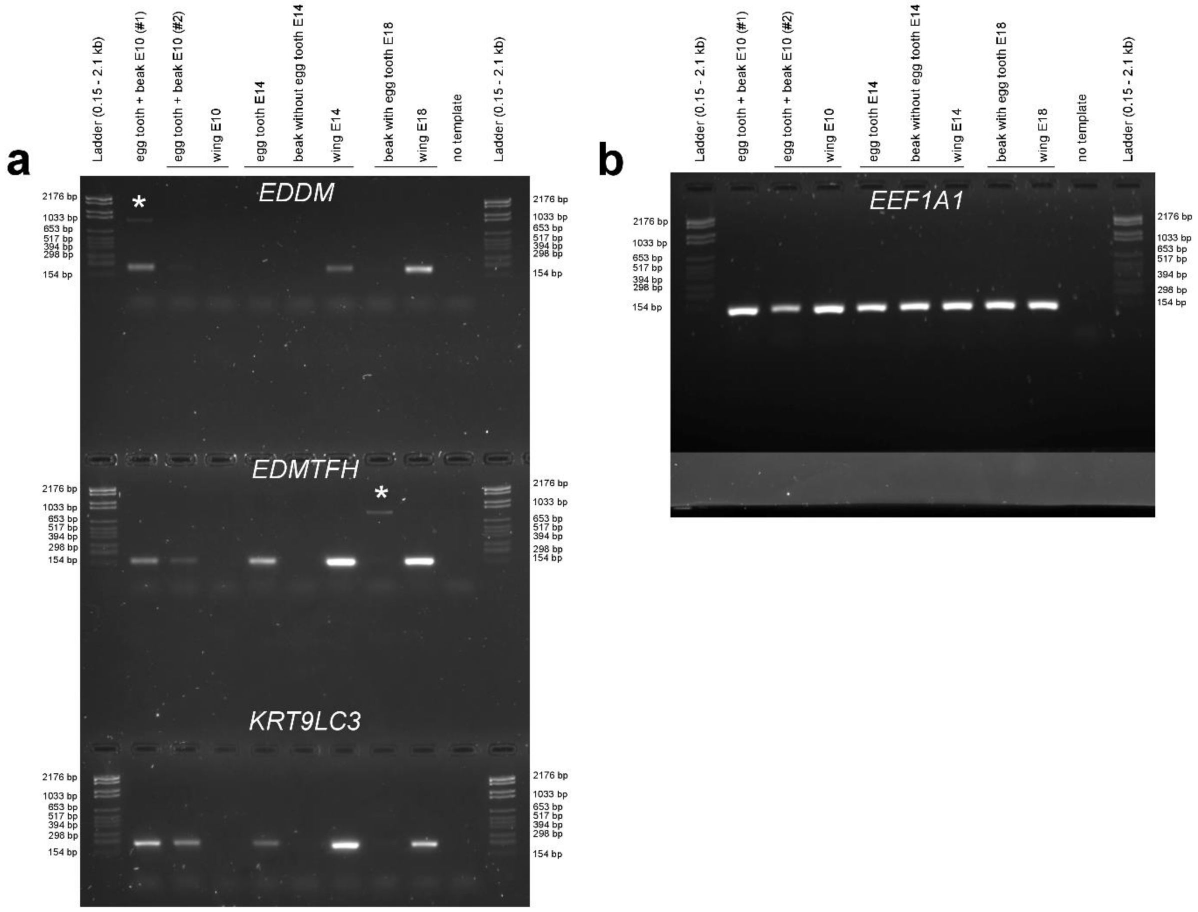

Figure S3. Whole-gel images corresponding to Figure 4. RT-PCR products of *EDDM*, *EDMTFH*, *KRT9LC3* (**a**) and of the house-keeping gene *EEF1A1* (**b**) were separated by agarose gel elecrophoresis. Asterisks indicate PCR products of genomic DNA (including intronic DNA). bp, base pairs.
